# Experimental Subarachnoid Hemorrhage in Rats: Comparison of Two Endovascular Perforation Techniques with Respect to Success Rate, Confounding Pathologies and Early Hippocampal Tissue Lesion Pattern

**DOI:** 10.1371/journal.pone.0123398

**Published:** 2015-04-13

**Authors:** Anke Höllig, Agnieszka Weinandy, Kay Nolte, Hans Clusmann, Rolf Rossaint, Mark Coburn

**Affiliations:** 1 Department of Neurosurgery, University RWTH Aachen, Aachen, Germany; 2 Department of Anesthesiology, University RWTH Aachen, Aachen, Germany; 3 Department of Neuropathology, University RWTH Aachen, Aachen, Germany; St Michael's Hospital, University of Toronto, CANADA

## Abstract

Recently aside from the “classic” endovascular monofilament perforation technique to induce experimental subarachnoid hemorrhage (SAH) a modification using a tungsten wire advanced through a guide tube has been described. We aim to assess both techniques for their success rate (induction of SAH without confounding pathologies) as primary endpoint. Further, the early tissue lesion pattern as evidence for early brain injury will be analyzed as secondary endpoint. Sprague Dawley rats (n=39) were randomly assigned to receive either Sham surgery (n=4), SAH using the “classic” technique (n=18) or using a modified technique (n=17). Course of intracranial pressure (ICP) and regional cerebral blood flow (rCBF) was analyzed; subsequent pathologies were documented either 6 or 24 h after SAH. Hippocampal tissue samples were analyzed via immunohistochemistry and western blotting. SAH-induction, regardless of confounding pathologies, was independent from type of technique (p=0.679). There was no significant difference concerning case fatality rate (classic: 40%; modified: 20%; p=0.213). Successful induction of SAH without collateral ICH or SDH was possible in 40% with the classic and in 86.7% with the modified technique (p=0.008). Peak ICP levels differed significantly between the two groups (classic: 94 +/- 23 mmHg; modified: 68 +/- 19 mmHg; p=0.003). Evidence of early cellular stress response and activation of apoptotic pathways 6 h after SAH was demonstrated. The extent of stress response is not dependent on type of technique. Both tested techniques successfully produce SAH including activation of an early stress response and apoptotic pathways in the hippocampal tissue. However, the induction of SAH with less confounding pathologies was more frequently achieved with the modified tungsten wire technique.

## Introduction

Despite extensive clinical and experimental research, aneurysmal subarachnoid hemorrhage (SAH) is associated with high morbidity and mortality rates [1–3]. SAH accounts for 5–7% of all strokes [4,5]. Often young and otherwise healthy persons are hit by this devastating disease. In addition to the individual fate caused by SAH it accounts for an enormous socioeconomic burden [6,7].

Until now the precise injury cascade and pathological mechanisms remain vague. Therefore experimental models are essential. Several preclinical studies reported promising results concerning ischemic and hemorrhagic stroke therapy, but the clinical translation remains inadequate. Hence, evaluation of experimental paradigms is essential to assure a proper basis for preclinical studies [8,9]. There are two major types of experimental SAH models in rats: The blood injection model [10,11], where blood is applied either into the cisterna magna or into the prechiasmatic cistern; and the endovascular perforation model [12,13]. Due to its similarity to human SAH the endovascular perforation model is regarded as the most suitable model despite its high mortality rates [12,14]. Recently, a modified endovascular perforation with tungsten wire instead of using a polypropylene filament has been described, which allows to perform a vessel perforation with a far smaller diameter. Limited data report large subarachnoid blood loads and a heterogeneous infarction pattern while undesired side effects and mortality rates were reduced [15,16]. We aim to assess the success rate of both endovascular perforation methods in terms of induction of SAH without collateral pathology as the primary objective. Secondary objective is clarification of the early hippocampal lesion pattern with respect to hippocampal stress response and activation of apoptotic signaling.

## Materials and Methods

The protocol for this study was approved by the government agency for animal use and protection (Protocol number: TVA 10416G1 approved by “Landesamt für Natur, Umwelt und Verbraucherschutz NRW” (LANUV), Recklinghausen, Germany), all experiments were performed in accordance with the Guide for the Care and Use of Laboratory Animals (National Research Council (US), and the Committee for the Update of the Guide for the Care and Use of Laboratory Animals; 8th edition, 2011).

### Animals

Male Sprague-Dawley rats (body weight 300–400 g, Charles River, Sulzfeld, Germany) were housed for at least one week before surgery with free access to food and water on a 12-hour light/dark cycle according to SPF (specific pathogen free) criteria. Animals were randomly (by lot) assigned for sham surgery (n = 4), traditional (classic) perforation technique (n = 18) or modified technique (n = 17). By drawing lots each rat was subjected to its survival time after SAH (either 6 or 24 h). The observation time of 6 h was chosen as one main secondary objective was the documentation of the very early brain injury. For additional information on mortality (in our experience mortality peaks between 6 and 24 h after SAH) a second observation time, namely 24 h, was scheduled.

Humane endpoints to sacrifice the animal prematurely were defined by assessing a score recommended by the local authorities of animal protection judging the general condition of the animal (including: 1. weight (0–20 points), 2. general condition including judgment of fur, appearance, tonicity (0–20 points), 3. behavior 80–20 points) and clinical status like body temperature, respiratory frequency (0–20 points)). Good general condition was equivalent to a score of zero points; the maximum score was 80 points. From 20 points on due to unacceptable extent of distress euthanasia was performed. The score was assessed at least twice daily by independent investigators. With respect to the distinct experimental procedure occurrence of seizures was added to the humane endpoints. Euthanasia was performed by exsanguination under deep anesthesia (see below) followed by decapitation.

### Anesthesia, Analgesia and Catheterization

Anesthesia was induced by intraperitoneal injection of Midazolam (2 mg/kg), medetomidine (0.15 mg/kg) and fentanyl (0.0075 mg/kg) [17,18]. For anesthesia maintenance a quarter of the initial portion was applied repeatedly (every 30 to 45 min via subcutaneous injection). Metamizole medication via intramuscular application was started for analgesic treatment directly after surgery (20 mg/kg every 8 h) and was maintained until euthanasia. The tail artery was used to insert an arterial line to measure blood pressure. Electrolytes and blood gases were monitored by recurrent arterial blood gas analysis. Body temperature was maintained at 37°C via a feedback-controlled heating pad (Physitemp Instruments, Inc., Clifton, New Jersey, USA).

### Surgical procedure

Regional cerebral blood flow (rCBF) bilaterally was measured continuously for the areas supplied by the middle cerebral artery via a two-channel laser Doppler flowmeter (Moor Instruments, Axminster, Devon, UK) as described previously [19]. In brief, a 1.5 cm midline incision of the skull in prone position was exposed and the bregma was identified. After thinning the bone with a water cooled highspeed drill 5.5 mm lateral (right and left) and 1 mm occipital of the bregma laser Doppler flow probes were attached to the skull. Continuous monitoring of intracranial pressure (ICP) was performed by placing an ICP-probe (Microsensor/ Codman ICP Express Monitor, Codman/ De Puy, Raynham, Massachusetts, USA) through a small parietal craniotomy.

Experiments started regularly 8 am in the same laboratory. After positioning the rat in a supine position, SAH was induced either by the “classic” polypropylene monofilament perforation technique initially described by Bederson et al. in 1995 and modified by Veelken et al. in the same year [12,13] or by the modified perforation method published by Park et al. [15]. SAH induction, via the classic technique, was performed using a 3–0 polypropylene suture with a diameter ranging from 200 to 250 μm (Prolene suture, Ethicon Inc., Somerville, NJ, USA). After exposing the left common carotid artery, the left internal carotid artery was identified and the suture was advanced as described in the literature. Perforation of the vessel and subsequent SAH was verified by a sharp rise of ICP as well as a drop of rCBF bilaterally.

SAH induction with the modified technique was carried out using a polytetrafluoroethylene (PTFE) tube with an outer diameter of 400 μm and an inner diameter of 200 μm (VWR, Radnor, Pennsylvania, USA), containing a tungsten wire with a diameter of 80 μm (Salomons Metalen, Groningen, NL). The PTFE tube was advanced through the left common carotid artery into the internal carotid artery and subsequently intracranially to the desired perforation area where the vessel perforation was performed by the tube-wire device (verified by drop of left rCBF). After induction of SAH the tube-wire was removed and the carotid artery was ligated. Of note, in the modified technique a far smaller filament-diameter (80 μm instead of 200–250 μm) is used.

In sham-operated animals the monofilament or the tube were advanced intracranially without perforation of the vessel.

Blood pressure (invasive measurement via tail artery catheter), rCBF, heart rate and ICP were recorded continuously 15 min before intervention (baseline), during intervention and 90 min thereafter with a special data acquisition system (PowerLab, ADInstruments, Spechbach, Germany).

### Assessment of blood load after SAH

High resolution photographs of the brains were obtained after having rinsed the brains with chilled isotonic solution to distinguish SDH from SAH. Images were analyzed according to the SAH bleeding scale for rats [20]. Basically, after dividing the basal cisterns into six segments, total scores were calculated based on a grading as follows:

Grade 0 = no SAH, grade 1 = minimal SAH, grade 2 = moderate SAH (vessels still visible through blood clot), grade 3 = massive blood clot (vessel no longer visible).

### Quantification of stress response and lesion pattern

Assessment of early lesion pattern after SAH was carried out by immunohistochemistry and protein analyses of the animals sacrificed 6 h after SAH. Brains were removed immediately after euthanasia. Blood load and occurrence of territorial, macroscopically visible territorial ischemia was documented by high resolution photography. Thereafter brains were divided into 2 mm coronal slices (1–7). Once again, photos of the slices were taken to allow documentation of intracranial hemorrhage (ICH) and ischemia, which later on was verified by histopathology. Slices were either embedded in paraformaldehyde for immunohistochemistry (coronal slice 5: 2 to 4 mm behind bregma), or hippocampal tissue (coronal slice 6: 4 to 6 mm behind bregma) was dissected for analysis of protein expression. Hippocampal tissue samples were snapfrozen and stored at -80°C.

### Lysate Preparation and Western Blot Analysis

Total protein content of hippocampal tissue was extracted for analysis using a commercially available RNA/Protein extraction kit (NucleoSpin RNA/Protein, Machery-Nagel, Düren, Germany). Western Blot (WB) analysis was carried out as described previously [21]. The following primary antibodies were used to probe the membrane:

Anti-Hypoxia-inducible factor 1-α (anti-HIF-1α, 1:1000; Abcam; Cambridge, UK), anti-cleaved Caspase 3 (1:1000; Cell Signaling Technology; Cambridge, UK) anti-cleaved PARP (1:1000; Cell Signaling Technology; Cambridge, UK) and anti-α-Tubulin (1:10.000; Sigma-Aldrich; St. Louis, MO, USA).

Horseradish peroxidase (HRP) conjugated goat anti-rabbit or anti-mouse (Thermo Scientific, Schwerte, Germany) as secondary antibody were used (dilution 1:5000).

Immunoreactivity was detected by enhanced chemiluminescence (Amersham Biosciences, Piscataway, NJ). Band intensity (normalized to α-Tubulin levels) was quantified using Adobe Photoshop CS5 (San Jose, CA, USA).

### Immunohistochemistry

Sections of 2 μm thickness were cut from paraffin embedded brain slices and placed on silane-coated slides, de-waxed, rehydrated and heated in citrate buffer for antigen retrieval. After blocking of non-specific binding by incubation in PBS containing 2% normal goat serum the slides were incubated for one hour (anti-NeuN), or over-night (anti-cleaved Caspase 3, anti-HIF-1α) with the primary antibodies diluted in blocking solution. Appropriate biotinylated secondary antibodies were used (1:200, Vector Laboratories Ltd., Peterborough, UK) for 15 min, followed by DAB visualization (DAKO, Carpinteria, CA, USA). Sections were photographed with the Axioplan microscope (ZEISS, Germany) using the Zen software (ZEISS, 20x and 40x magnification). The region of interest was defined as left hippocampus (ipsilateral to SAH induction).

### Quantification and Statistical Analysis

Power analysis was carried out prior to the start of experiments. Based on previously published data demonstrating a success rate of 48% with the classic vs. 90% with the modified method [15] the estimated sample size (for differentiation of success rate) was calculated with n = 14 (power: 1-β = 0.8; α = 0.05). As dropouts (due to anesthetic or procedural complications) were expected, we included at least n = 17 per group. Success rate was defined as successful induction of SAH without comorbidities (ICH, SDH or ischemia). Secondary objectives (protein analysis, immunohistochemistry) were assessed by independent investigators. Categorical variables for statistical analysis were compared via Chi square test between the two groups (classic vs. modified group). When the expected values in any of the cells of a contingency table were below five, one-tailed Fisher´s exact test was used. Independent t-test was applied for metric values. Charts of rCBF and ICP are given with means ± SD (standard deviation). The data measured via semiquantitative analysis of protein expression and immunoreactivity from three independent experiments was expressed as means ± SD. All calculations were performed using SPSS 21.0. Statistical significance was accepted with a p- value <0.05. Significance is indicated with (***) p<0.001, (**) p<0.01 and (*) p<0.05; (#) indicates no significant difference.

## Results

### Baseline parameters

Microbiological testing did not reveal evidence for infection in any animal. Baseline weight of the animals (classic versus modified group) did not differ significantly (342 ± 22 versus 342 ± 29 g, p = .969; independent t-Test). All animals were included for analysis.

### Success rate and collateral damage

**Fig 1:**
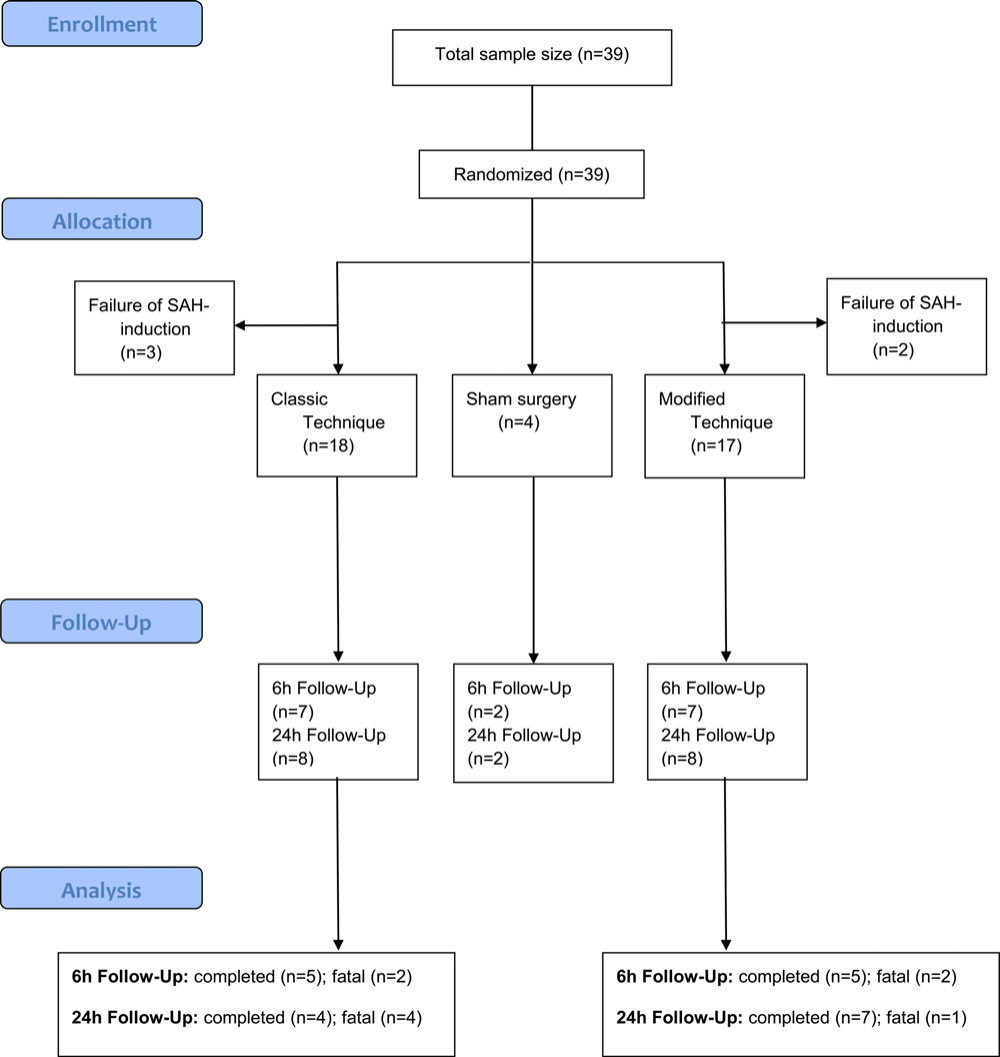
Diagram of animals enrolled. Diagram of animals enrolled: total sample size n = 39, invalid rCBF-data in n = 3 (6 h modified technique n = 1; 24 h modified technique n = 2).

39 animals were analyzed, from which n = 18 were randomly assigned to receive SAH by classic endovascular perforation technique (classic group) and n = 17 by modified endovascular perforation technique (modified group) (Fig 1). Due to unsuccessful induction of SAH 5 animals (classic group: n = 3; modified group: n = 2) had to be excluded (Fig 1). Sham surgery was performed in n = 4 cases. Success rate of intervention (total: 85.6%) with regard to SAH induction was similar for both groups indicating a failure rate of 16.7% with the classic technique and 11.8% with the modified technique (p = 0.528; one-tailed Fisher´s exact test). The classic technique involved a case fatality of 40% (n = 6), whereas induction of SAH via the modified technique resulted in a case fatality rate of 20% (n = 3; p = 0.213; one-tailed Fisher´s exact test). The case fatality rate of both observation time groups (regardless from technique of induction) did not differ significantly (p = 0.596; one-tailed Fisher´s exact test). Comparing the case fatality rate of each time group (6 and 24 h after SAH) with respect to technique of SAH induction no significant difference was found (6 h: p = 0.406; 24 h: p = 0.365; one-tailed Fisher´s exact test).

Relevant ischemia was considered as a territorial cerebral infarction visible macroscopically on photo documentation and confirmed by histopathological examination. Defined as such, occurrence of ischemia was seen in 53% (n = 8) of the animals suffering from SAH induced by classic technique. Despite a trend of lower incidence of ischemic events (33%; n = 6) using the modified technique, statistical significance was not achieved (p = 0.231; one-tailed Fisher´s exact test). Noticeably, ischemic events were mainly visible in fatal cases (7 out of 9).

Examining the brains of fatal cases retrospectively, most probably the cases of death were a result of increase of intracranial pressure due to global brain swelling accompanied by territorial ischemia.

Collateral intracranial hemorrhages, like intracerebral (ICH) or subdural hematoma (SDH), were detected in a considerable number of cases. Irrigation with chilled isotonic solution allowed distinction of SDH versus SAH. In summary, a significant difference between the two methods could be detected: ICH was observed in 26.7% (n = 4) of the classic group, whereas none were found in the modified group (p = 0.050; one-tailed Fisher´s exact test). Similarly, incidence of SDH varied between the two groups (classic technique: 46.7%; modified technique: 13.3%), but barely reached significance (p = 0.054; one-tailed Fisher´s exact test). Induction of SAH without collateral ICH or SDH was possible in 40% with the classic and in 86.7% with the modified technique (p = 0.008; Chi square).

### Intracranial pressure and cerebral blood flow

**Fig 2:**
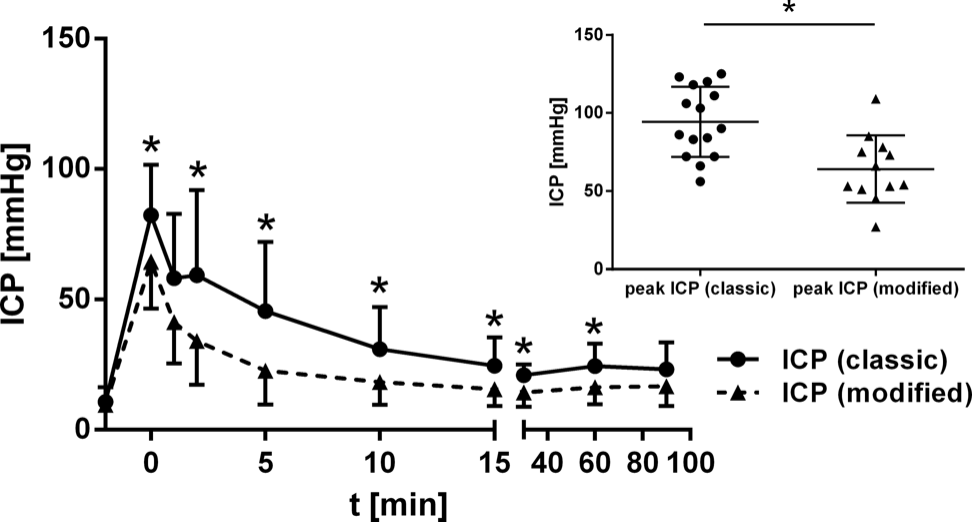
Chart for course of ICP. Course of ICP (mean +/- standard deviation) dependent on technique, * p<0.05, Boxplot of peak ICP (* p<0.05).

Peak ICP levels differed significantly between the two groups (mean ICP +/-SD: classic technique: 94 +/- 23 mmHg; modified technique: 68 +/- 19 mmHg; p = 0.003; independent t-Test, Fig 2). ICP levels measured repetitively during the experimental course were notably distinct between the two techniques reaching statistical significance between 2 and 60 min after induction of SAH (p<0.05; independent t-Test; Fig 2). However, a trend for higher ICP levels with the classic technique was already observed 1 min after SAH induction (p = 0.062; independent t-Test). Baseline ICP levels did not vary substantially (classic technique: 11 +/- 6 mmHg; modified technique: 10 +/- 2 mmHg; p = 0.550; independent t-Test).

**Fig 3:**
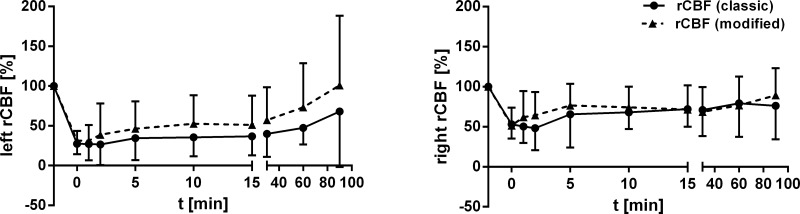
Course of left and right rCBF. Graphs showing percentage changes (mean +/- standard deviation) of left and right rCBF (percentage of baseline).

Concerning course of CBF, a notable distinction between the groups was not observed (left rCBF: p = 0.446; right rCBF: p = 0.857; independent t-Test; Fig 3).

**Fig 4:**
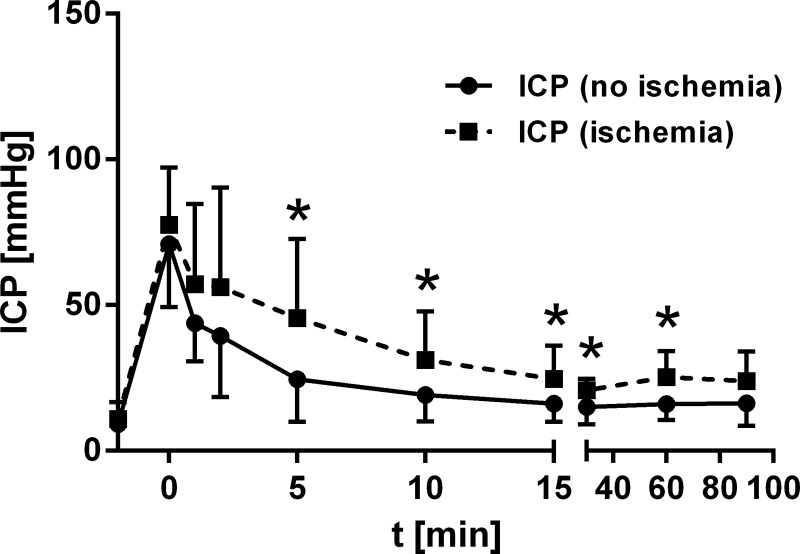
Course of ICP dependent on occurrence of territorial ischemia. ICP-levels (5 min to 90 min after SAH) differ significantly dependent on occurrence of ischemia (* p<0.05) with higher ICP-levels observed in cases with territorial ischemia.

Interestingly, in the subgroup with collateral ischemia from both the classic and the modified group, ICP levels measured between 5 and 60 min after SAH induction were significantly higher compared to animals without ischemia (p<0.05; independent t-Test; Fig 4). However, despite a proportion of 62% induced via classic technique in the group with ischemia, the peak ICP level was similar between these two groups (with vs. without ischemia: p = 0.428; independent t-Test), but from 5 min after SAH on significance was demonstrated (Fig 4).

### Hippocampal stress response and lesion pattern 6 h after SAH

Equal blood loads 6 h after SAH induction (according to bleeding scale published by Sugawara et. al [20]) were shown independent from type of technique (p = 0.368; independent t-Test).

**Fig 5:**
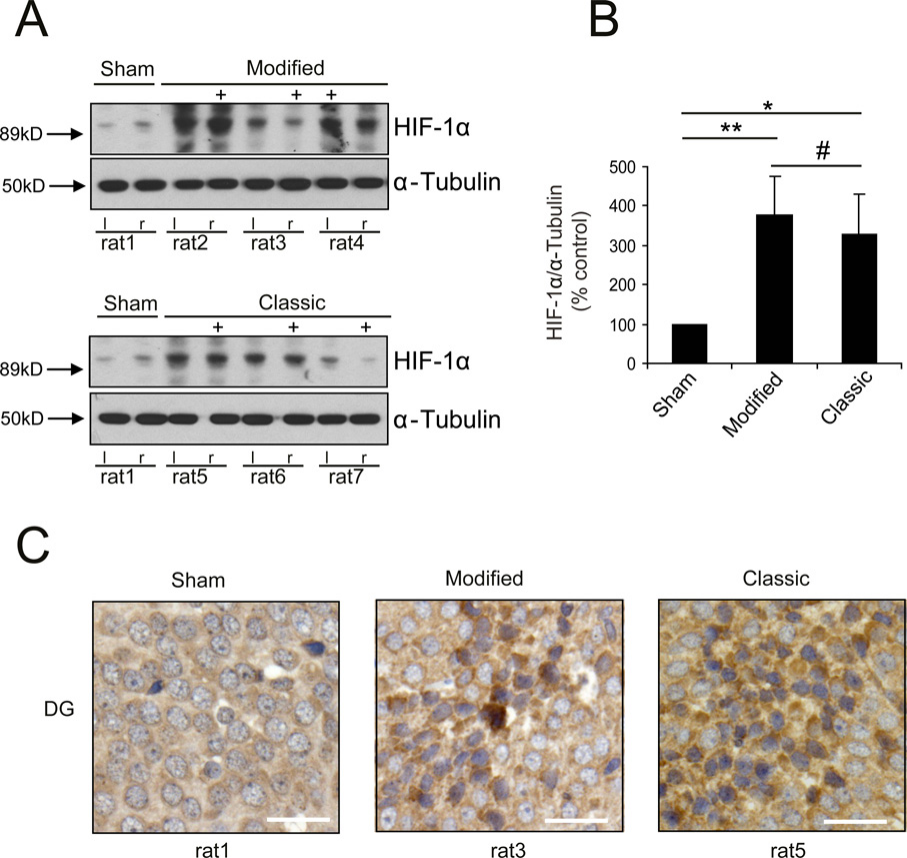
Analysis of HIF-1α-expression. Protein analysis for HIF-1α shows significant increase of HIF-1α-expression in hippocampal tissue 6h after SAH (A+B) independent from type of technique (Location of ICP-probe is hallmarked (+)). Immunohistochemical staining for HIF-1α demonstrates clear increase of HIF-1α expression in dentate gyrus compared to Sham animal (right lower corner: bar indicates 20 μm). (** p<0.01; * p<0.05; # p>0.05)

Hypoxia inducible factor 1α (HIF-1α) is a transcription factor which is known to be activated after SAH. Using protein analysis we were able to show an increase of HIF-1α-expression in hippocampal tissue after experimental SAH independent from type of technique (Fig 5A). Levels of protein expression contralateral to ICP probe were semiquantified, demonstrating a marked difference of hippocampal HIF-1α expression between tissue samples of SAH-animals (both techniques) and Sham animals (p<0.05; Fig 5B). Immunohistochemical staining of left hippocampal area for HIF-1α (Fig 5C) endorses these findings.

**Fig 6:**
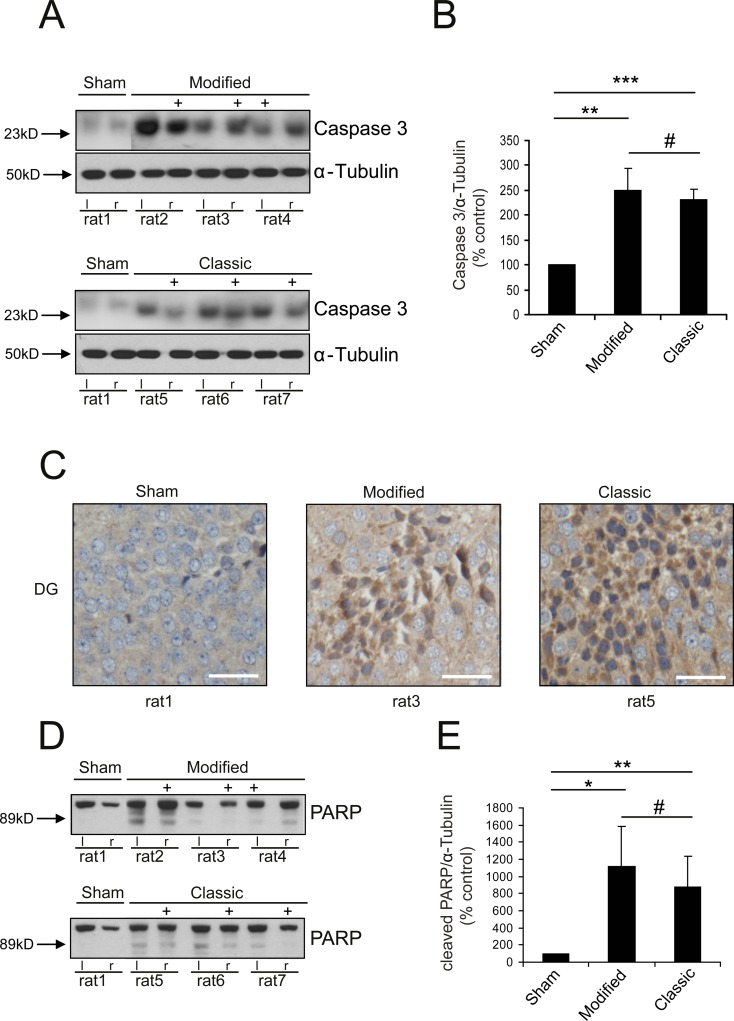
Induction of apoptotic signaling. Western Blots show an increase of caspase 3 expression (A) and PARP cleavage (D) in SAH animals (Location of ICP-probe is hallmarked (+)). Immunohistochemical staining for caspase 3 (C) confirms these results (right lower corner: bar indicates 20 μm). Semiquantitative analysis of protein expression demonstrates significant increase of caspase 3 (B) and PARP cleavage (E) in SAH animals (independent from technique). (*** p<0.001; ** p<0.01; * p<0.05; # p>0.05)

Poly ADP-ribose polymerase (PARP) is a nuclear protein involved in cellular stress response mediating both neuronal death and survival [22]. Cleavage of PARP by caspases (cysteine-aspartic acid proteases) is a typical sign of apoptosis, which can be observed in the context of many neurological diseases, e.g. cerebral ischemia or Parkinson´s disease [23,24]. In vivo caspase 3 seems to be mainly responsible for PARP cleavage [25]. We were able to show an increase of caspase 3 expression by western blotting hippocampal tissue samples at an early stage (6 h) after SAH, independent of technique compared to Sham animals (Fig 6A and 6B). Additionally, cleaved PARP fragments in hippocampal tissue of SAH animals were significantly increased compared to Sham animals indicating apoptotic signaling (p<0.05; Fig 6D and 6E). Again, no significant difference could be observed between the two techniques. Immunohistochemical analysis by caspase 3 staining confirms the results from western blotting (Fig 6C).

**Fig 7:**
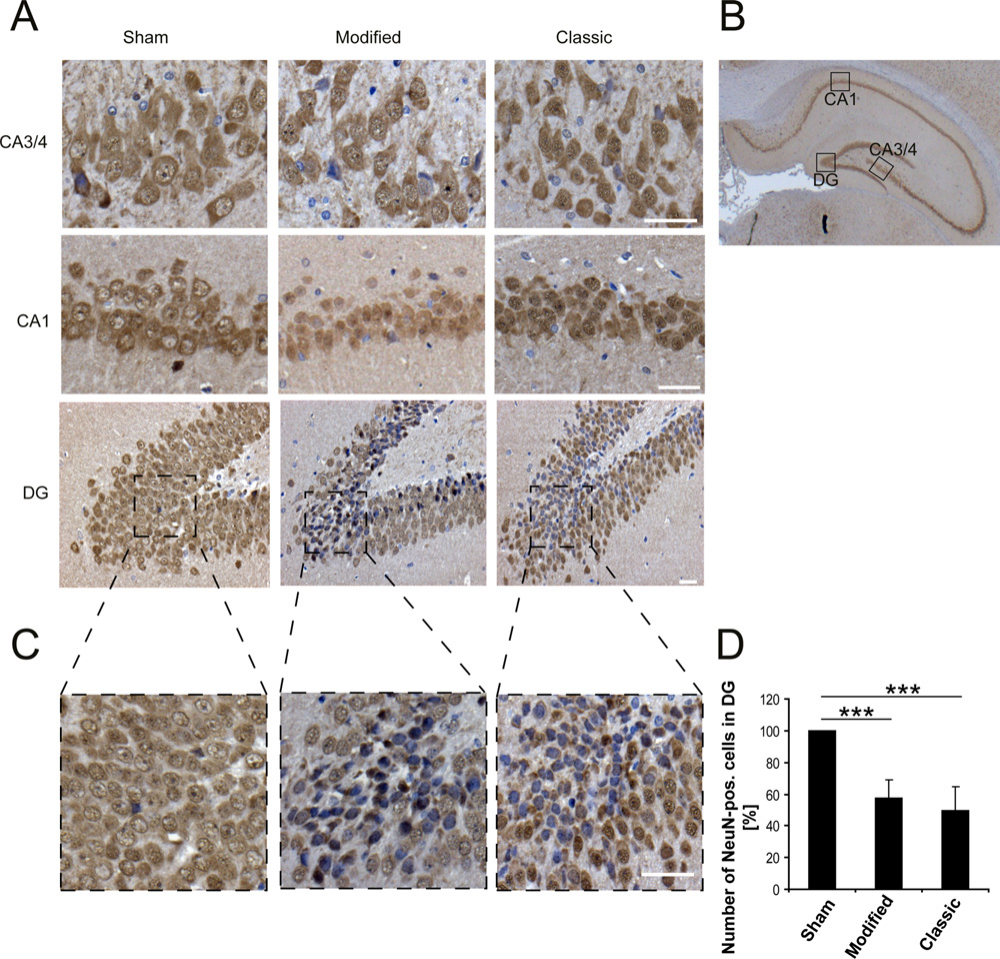
NeuN staining for 3 different hippocampal regions. Graph shows decrease of NeuN staining for 3 hippocampal regions (CA3/4, CA1 and dentate gyrus) 6 h after SAH independent from technique compared to sham surgery (A). Decrease of NeuN immunoreactivity predominantly is seen in dentate gyrus and significantly reduced 6 h after SAH (right lower corner: bar indicates 20 μm). (*** p<0.001)

NeuN, a neuron-specific nuclear protein, is a common biomarker for neurons. Immunohistochemical staining for NeuN demonstrated a decrease of hippocampal immunoreactivity 6 h after SAH predominantly in the dentate gyrus (DG) regardless of technique (Fig 7).

## Discussion

Both the classic endovascular filament perforation model and the modified tube-tungsten wire technique, which allows perforation with a far smaller diameter (80 μm), produce SAH successfully. However, our primary endpoint, the induction of SAH without confounding pathologies, was met more reliably by the modified technique. With the classic technique, significantly higher levels of peak ICP are reached. Furthermore, the modified technique seems favorable with respect to lower mortality rate and reduced confounding pathologies. Activation of stress response and apoptotic pathways in hippocampal tissue samples was observed with both techniques during the early course of SAH.

The results of this study are similar to the reported observations from Park et al. introducing the modified technique in 2008 [15]. By a proof-of-principle study decrease of mortality rate (46 to 19%), increase of success rate (48 vs. 90%) and reduced occurrence of subdural hematomas (SDH, 44 vs. 6%) was demonstrated by Park et al. [15]. Hence, our results (even if not statistically significant) support the impression of decreased mortality and reduced occurrence of SDH by the modified technique. Thus, providing a disease model which includes less confounding factors (like SDH or ICH) but also the pathophysiological similarity to human SAH preclinical studies may deal with a simplified (no ICH as possible with human SAH), but also more reproducible setting compared to the classic model. As a first step, examining neuroprotective strategies in the context of SAH without confounding pathologies this may facilitate an iterative approach to investigate the pathophysiology of SAH. Promising preclinical results surprisingly often fail when translated into clinics. Stroke research has shown that one aspect of preclinical therapeutic strategies failing in clinics at least may be the use of inappropriate animal models [26–28]. Same applies for research in the context of SAH. Multiple models are exerted, each of them owing similarities as well as differences compared to human SAH. Thus, this model may offer a first step for research providing reliable induction of mere SAH with low mortality rate and rare occurrence of confounding pathologies.

In contrast to the approach of Park et al. a 3–0 polypropylene suture was chosen in accordance with other studies of same sized animals [13,29]. Appropriately, a slightly thicker PTFE tube (400 μm outer diameter instead of 300μm) was used. Probably due to this fact peak ICP levels reached by the modified technique used in our study are substantially higher than previously published. Differences in the mortality rates may arise from the different time points of assessment (6 respectively 24 h after SAH induction in our study vs. 48 h after SAH in the study by Park et al.).

Neuronal damage in the course of SAH induced by modified technique has already been described by Greenhalgh et al. [16]. In this work neuronal loss and breakdown of blood brain barrier 24 h after SAH had been assessed by histological and immunohistochemical investigations. Using the modified technique we additionally demonstrate detectable signs of early brain injury (6 h after SAH):

Recently research efforts have shifted more and more towards the investigation of the early lesion mechanisms after SAH, the so-called early brain injury (EBI) [30–32]. To address these early mechanisms in SAH-pathology it is crucial that the experimental setting properly reflects the desired lesion pattern.

Activation of caspase 3 has been shown as early as 10 min after SAH [33]. Even if not always resulting in cell death caspase 3 activation indicates hampered cell function. We were able to confirm the finding of early stress response and activation of apoptotic signaling 6 h after SAH (indicated by increase of caspase 3 expression and cleaved PARP fragments) using the classic as wells as the modified technique. The extent of the stress response and the activation of apoptotic signaling in our study were independent from type of technique.

Cleavage of PARP in the hippocampal tissue after SAH so far not has been shown in vivo. At present, PARP as a modulator of inflammation mainly has been implicated in vasospasm after SAH [34,35]. In vitro, oxyhemoglobin induces PARP cleavage in astrocytes and smooth muscle cells resulting in necrosis [36,37]. For cerebral endothelial cell this has also been demonstrated [38]. Here, we present for the first time hippocampal PARP cleavage as a molecular alteration after experimental SAH.

He observed decrease of neuron-specific nuclear protein (NeuN) immunoreactivity 6 h after SAH is likely to indicate incipient cell death. However, hippocampal neuronal loss was not shown probably due to the very early examination. Cell death has been previously shown by identification of degenerated neurons via Fluoro-Jade staining from 24 h after SAH on [33].

HIF-1α as a key regulator following cerebral ischemia is activated after SAH. It is. However, it is somewhat ambiguous, whether protective or deleterious effects arise from the activation of HIF-1α after SAH [39–41]. Different observation times may have confounded conclusions drawn in the literature. Here, we demonstrate that activation of HIF-1α (independent from the perforation technique) occurs within 6 h after SAH.

Additionally to previous studies reporting small areas of infarction we observed territorial ischemia (predominantly in the classic group) [16]. Similar to the hypothesis provided by Park et al., we suppose that the classic technique may produce stretching of the ICA and therefore a slight dislocation relating to the skull base so that perforation occurs directly at the skull base entrance, regardless of a sharpened or blunt tip of the polypropylene suture. With this probable subdural perforation of the vessel SDH is created instead of SAH. The course of experiments in our hands supports this theory; similar to the classic technique the feeling of stiffness or resistance was observed in those cases, when SDH was diagnosed thereafter. Basically, due to the large diameter of the polypropylene suture (4–0: 200 μm to 2–0: 300 μm) compared to the diameter of the rat ICA, MCA and ACA (300–350 μm; 240 μm and 280 μm) [15,42] with the classic technique-in our impression- the target vessel is disrupted rather than perforated. In our study a rather thick polypropylene suture (3–0) was used, which most likely contributes to the frequency of territorial ischemia. These facts may explain the astonishingly high number of ischemic events in the classic group which, to our knowledge, has not been published before. Additionally, territorial ischemia occurred predominantly in the fatal group, which is usually not incorporated into analysis of outcome measurements and therefore probably not reported in publications.

As aforementioned, various differences between the two techniques may mainly reflect the difference in perforation diameter. It has already been demonstrated that the filament diameter influences the peak ICP as well as distribution of blood load [43]. The modified technique allows the usage of a filament with a far smaller diameter advanced through a guide tube. Thus, it rather resembles the possibility to perform a smaller perforation than a completely distinct technique.

Limitations of the study arise from small group size calculated to assess the success rate of the methods as primary endpoint. Therefore, statistical significance of characteristics like mortality and occurrence of subdural hematoma was not reached. Another possible disadvantage of this study is the high rate of ischemia after intervention. We attempted to minimize the possibility of a technical error as source of ischemia frequency, such as hesitant advancement of suture resulting in transient occlusion of the MCA. This possibility could be ruled out by analysis of CBF: A sharp rise of ICP occurred directly after left CBF drop. With respect to the heretofore-mentioned anatomical and specific experimental condition, we assume that occurrence of ischemia may be dependent on the distinct methodical and anatomical circumstances and play a role in the high mortality rate with the classic technique.

Due to application of the ICP probe, unilateral hippocampal traumatization occurs. Therefore, protein analysis of hippocampal tissue is somehow biased by these lesions as only hippocampal tissue contralateral to ICP probe can be examined.

Additionally, the left internal carotid artery (ICA) in our model has been ligated. It may be speculated that the high incidence of ischemic event results from hypoperfusion prior to SAH. Anyways, a relevant drop of CBF after ligating the ICA with our monitoring has not been observed, rather a slight decline returning nearly to baseline values after some seconds.

In summary, the modified endovascular perforation technique with tube and tungsten wire seems to bring major advantages compared to the classic technique. Although peak ICP reached by the model is significantly lower than that of the classic technique, equal drop of rCBF and early stress response can be achieved making it suitable for examining the pathophysiology of early brain injury after SAH. Moreover a more specific induction of SAH and a lower mortality rate exceeds the disadvantages of the method. Besides, since the number of animals can be reduced ethical concerns and cost issues also favor of the modified technique.
